# Analysis of highly polar anionic pesticides in food of plant and animal origin by ion chromatography and tandem mass spectrometry with emphasis on addressing adverse effects caused by matrix co-extractives

**DOI:** 10.1007/s00216-024-05389-4

**Published:** 2024-06-19

**Authors:** Ann-Kathrin Schäfer, Walter Vetter, Michelangelo Anastassiades

**Affiliations:** 1 Section of Residues and Contaminants, Chemisches und Veterinäruntersuchungsamt Stuttgart, Fellbach, D-70736 Germany; 2 Institute of Food Chemistry (170b), University of Hohenheim, Stuttgart, D-70599 Germany

**Keywords:** Polar pesticide, Ion chromatography, Tandem mass spectrometry, IC-MS/MS, Matrix effect, Glyphosate

## Abstract

Residues of various highly polar pesticides and their metabolites are commonly found in numerous food products. Some of these compounds, such as glyphosate, are not only used in large amounts in agriculture, but are also controversially discussed in public. Here, we present a method, employing ion chromatography (IC) coupled to tandem mass spectrometry (IC-MS/MS), for the analyses of glyphosate, aminomethyl phosphonic acid (AMPA), *N*-acetyl-glyphosate (NAGly), fosetyl, and 10 further highly polar pesticides and metabolites in various plant and animal matrices following a minimal sample preparation by means of the QuPPe method. Thorough investigations showed that an AS19 column enabled the analysis of all 14 compounds within 30 min. The best sensitivity could be obtained with the make-up solvent acetonitrile being admixed to the mobile phase at a 1:2 flow rate ratio. Matrix effects were thoroughly studied in terms of ion suppression and retention time shifts. Conductivity detection was used to monitor elution profiles of matrix co-extractives in comparison with matrix effect profiles obtained by continuous post-column infusion of a mix with 13 highly polar pesticides and metabolites. These tests indicated that a fivefold dilution of QuPPe extracts was suitable for the routine analysis of samples for MRL-conformity, as it considerably reduced matrix effects maintaining sufficient sensitivity and high recovery rates in eight different commodities. The suitability of the final method for its application in routine analysis was verified by the analysis of >130 samples containing incurred residues where the results were compared with two existing LC-MS/MS methods.

**Graphical Abstract:**

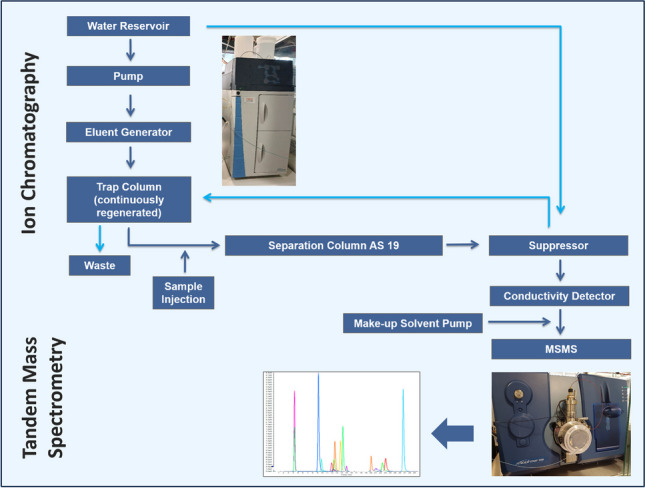

**Supplementary Information:**

The online version contains supplementary material available at 10.1007/s00216-024-05389-4.

## Introduction

In modern agriculture, pesticide formulations are widely used to maintain high crop yields. The many active substances used differ remarkably in their physicochemical properties, which range from high lipophilicity [1] to high hydrophilicity (polarity) [2, 3]. Some of the very polar pesticides, i.e., the total herbicides glyphosate and glufosinate along with the fungicide phosphonic acid, are currently among the most commonly used pesticides in agriculture [4]. The plant growth regulator ethephon is another highly polar compound used in many crops, such as cereals, pineapples, tomatoes, figs, apples, and cherries. At the same time, however, concerns about the safety of some of these highly polar agrochemicals have led to calls for a ban [5].

Due to their high polarity, these analytes are not amenable to the QuEChERS method [6, 7], i.e., the most common multi-residue method in the area of pesticide residue analysis [8, 9]. Former strategies for determining glyphosate and other highly polar analytes involved their derivatization in order to make them amenable to gas chromatography (GC) with mass spectrometry (MS) [10, 11], nitrogen-phosphorus detection (NPD), or the flame photometric detection (FPD) [12]. Likewise, reversed-phase high-performance liquid chromatography in combination with fluorescence detection (HPLC-FLD) [13] or tandem mass spectrometry (LC-MS/MS) [14, 15] has been used for the determination of certain derivatives. However, methods involving derivatization steps are limited in their applicability by the fact that not all highly polar analytes can be derivatized with the same agent. For instance, the popular agent fluorenylmethyloxycarbonyl chloride (FMOC-Cl) allows to derivatize primary or secondary amino groups, but not analytes without an amino group such as the organophosphorus compounds (e.g., ethephon, fosetyl, and phosphonic acid) as well as *N*-acetylated metabolites. A viable alternative is the quick polar pesticides (QuPPe) method, which enables the direct LC-MS/MS measurement of polar pesticides without derivatization [16].

The QuPPe method is based on an extraction with acidified methanol and includes only marginal clean-up. Hence, the resulting extracts typically contain different types of polar co-extractives, such as organic acids, soluble proteins, and sugars [17, 18]. These co-extracted matrix compounds may impair both the chromatographic and ionization performance in form of retention time (*t*_R_) shifts and ion suppression of affected analytes during LC-MS/MS analysis with electrospray ionization (ESI). In the case of glyphosate, its main metabolite aminomethylphosphonic acid (AMPA), and some other highly polar analytes, signal suppressions in LC-MS/MS sometimes exceed 80%, compromising sensitivity and increasing the risk of false negative results [19].

As an alternative, ion chromatography (IC) has been used for the analysis of polar pesticides, since the separation is based on a different approach compared to LC-MS/MS, thus partly providing a better separation of polar pesticides from problematic matrix compounds [16, 20–22]. For instance, IC coupled to tandem mass spectrometry (IC-MS/MS) or Orbitrap high-resolution mass spectrometry (HRMS) has been successfully used for the determination of different analytes in different vegetables matrices [20, 23, 24] (see also brief comparison in Table S1). In these studies, methanol, 2-propanol, and acetonitrile (ACN) were used as make-up solvents via post-column infusion to increase ionization yields [20, 23, 24]. Other approaches used IC phases designed for elution with bicarbonate in water-ACN that were employed in a LC system coupled to MS/MS [21, 22].

The goal of the present work was the development of a method for the IC-MS/MS analysis of glyphosate, glufosinate, ethephon, and 11 further highly polar anionic analytes after sample preparation by the QuPPe method. Since matrix effects are known to vary considerably from matrix to matrix, a variety of commodities of plant and animal origin was studied, including fruits, vegetables, pulses, cereals, and liver and milk. The investigations included the testing of two promising IC columns along with the type and the relative flow rate of the make-up solvents. To better study the matrix effects, these were visualized via post-column infusion of pesticide mixes in different problematic matrices. Considering the impact of extract dilution on the degree of the matrix effects and the overall sensitivity of the method, appropriate dilution factors of QuPPe extracts prior to the IC-MS/MS analysis were established. Finally, the results obtained using the validated IC-MS/MS method were compared with those obtained by two established LC-MS/MS methods.

## Experimental

### Chemicals and samples

Methanol, ACN, and 2-propanol (MS grade, respectively) were purchased from either Merck/Supelco (Darmstadt, Germany), Th. Geyer (Renningen, Germany), or Biosolve Chemicals (Valkenswaard, The Netherlands) (different sources due to supply difficulties). Formic acid was purchased from Honeywell (Charlotte, NC, USA). Ethylenediaminetetraacetic acid tetrasodium salt tetrahydrate (Na_4_ EDTA × 4 H_2_O) was purchased from Merck/Millipore (Darmstadt, Germany), while C_18_ sorbent (POLYGOPREP 300-30 C18, particle size 30 µm, pore size 300 Å) was ordered from Macherey-Nagel (Düren, Germany). Standard substances of organic acids were purchased from Merck/Sigma-Aldrich (Darmstadt, Germany). The matrices and samples used for experiments and method validation were collected from the local market in organic quality. Exceptions were Swiss chard and rhubarb, which were grown in a private garden close to Stuttgart (Germany). Ultrapure water for instrumental analysis was prepared from pure water using a Millipore Milli-Q IQ 7000 system (Darmstadt, Germany).

Standard substances of native analytes and isotopically labeled internal standards (IL-ISs) were purchased from Dr. Ehrenstorfer (Augsburg, Germany), LGC/Toronto Research Chemical (TRC, Teddington, England), HPC (Cunnersdorf, Germany), Merck/Sigma-Aldrich (Darmstadt, Germany), Merck (Darmstadt, Germany), ASCA (Adlershof, Germany), and LGC/Fluka (Teddington, England). Most IL-ISs were purchased from TRC while the IL-ISs of chlorate, perchlorate, and phosphonic acid were synthesized in our laboratory (Table S2, supplementary material). Stock solutions were prepared in water containing 10% ACN at concentrations of 1000 µg/mL with the following exceptions: Stock solutions of fosetyl and its IL-IS were prepared in the same solvent mixture but only at 100 µg/mL due to solubility issues. Stock solutions of ethephon and its IL-IS were prepared in 10% ACN in water containing 0.1% HCl, as ethephon is base-sensitive. These stock solutions were used to prepare mixtures at various analyte concentrations (e.g., 10 µg/mL) by diluting with 10% ACN in water. The residual acid after dilution was still sufficiently protective towards ethephon.

### Instrumentation

The IC system consisted of a Dionex Integrion HPIC instrument equipped with an AS-AP autosampler (Thermo Fisher Scientific, Waltham, MA, USA) and interfaced to an AB Sciex QTrap 5500 mass spectrometer (AB Sciex, Framingham, MA, USA). Analyses were performed with electrospray ionization (ESI). The IC was monitored with Chromeleon software (Thermo Fisher Scientific, Waltham, MA, USA) and the MS with Analyst software (AB Sciex, Framingham, MA, USA). An AXP-MS auxiliary pump (Thermo Fisher Scientific Dionex, Waltham, MA, USA) was used as a make-up solvent pump. An additional external peristaltic/tubing pump, the Reglo Digital (Ismatec/Cole-Parmer, Vernon Hills, IL, USA), was used for the continuous regeneration of the suppressor. Two 250 mm length × 2 mm internal diameter analytical columns (AS19 and AS24, Thermo Fisher Scientific Dionex, Waltham, MA, USA) were used in combination with respective pre-columns. Both stationary phases are based on polymeric materials entailing quaternary ammonium moieties as ion exchange groups, with the AS24 showing a higher ion exchange capacity and a lower hydrophobicity. The IC flow rate was set at 0.3 mL/min and the KOH concentration of the eluent started and was kept for 8 min at 15 mM KOH and was then increased to 36 mM KOH within 5 min and was held for 8 min; then increased to 70 mM KOH within 0.5 min and held for 3.5 min [16, 25]. For re-equilibration, the KOH concentration was reduced to 15 mM KOH at 25.5 min and held for 4.5 min [16, 25]. The injection volume was 5 µL of fivefold diluted extracts and the column temperature was set at 32 °C [16, 25]. A Dionex ASRS 300 2 mm (Thermo Fisher Scientific Dionex, Waltham, MA, USA) was used as a suppressor (Table S3, supplementary material). Two, or where possible three, mass transitions were recorded for each standard substance and one transition for each IL-IS (Table S2 and Table S4).

For comparing validation data with LC-MS/MS, the following systems were used in ESI mode: LC-MS/MS with a porous graphitized carbon (PGC; Hypercarb) column: Agilent Technologies 1290 Infinity II (Agilent, Santa Clara, CA, USA) in combination with an AB Sciex QTrap 6500^+^ system. LC-MS/MS HILIC (Torus DEA): Waters Acquity UPLC I-Class (Waters, Milford, CT, USA) in combination with an AB Sciex QTrap 5500 instrument (parameters are shown in Table S3). LC-MS/MS chromatograms of a mix of ten pesticides are shown in Fig. S1.

### Sample preparation

Samples of plant origin (PO) were prepared according to the QuPPe-PO method [16]. In brief, samples (5 or 10 g) were extracted with acidified methanol (containing 1% formic acid), followed by mechanical shaking, a freeze-out step, centrifugation, and filtration [16]. To increase recovery rates of glyphosate and its metabolites and to remove proteins from the sample extract, the QuPPe extraction method was modified for matrices containing high amounts of protein and/or lipids, here: soybean, sesame, rice. In these cases, the extraction procedure was complemented by the use of EDTA and a clean-up step during which the extract was diluted with ACN (1:1) and subjected to a dispersive SPE (dSPE) step using octadecylsilane (ODS) sorbent, with the purpose of removing lipids and proteins [16, 26]. Samples of animal origin (AO) were processed with the QuPPe-AO method [26], which essentially resembles the abovementioned procedure involving the use of EDTA during extraction and clean-up by dSPE (ODS) of the ACN-diluted extract [26]. Blank matrix extracts (for studying matrix effects) were prepared on a residue-free matrix (without detectable levels of any of the analytes of interest), with the exception of phosphonic acid, which was contained at trace levels and HEPA, which is suspected to be naturally formed in bovine intestines and was encountered in bovine liver [27]. No internal standards were added during extraction to obtain the blank extracts. Further modifications will be described in each experiment.

### Improving the ESI signal intensity by adding an organic make-up solvent

To facilitate evaporation and ionization in the ESI source, organic solvents (methanol, ACN, or 2-propanol) were added to the originally fully aqueous IC effluent in defined percentages using an external pump with suitable flow rates (see below). Methanol, ACN, or 2-propanol was infused via a T-piece between the conductivity detector (CD) of the IC system and the MS/MS ion source. In this experiment, a standard mixture of the analytes in pure water at 0.01 µg/mL was injected three times using the AS19 column, and the average peak areas obtained were compared to each other. Specifically, the IC flow rate was kept constant at 0.3 mL/min, and the make-up solvents methanol, ACN, or 2-propanol were individually added at a ratio of ~1:4 (0.08 mL/min), 2:4 (0.15 mL/min), ~3:4 (0.23 mL/min), 4:4 (0.3 mL/min), and ~5:4 (0.38 mL/min). All experiments were carried out in triplicate (*n*=3), and mean values and relative standard deviations (RSD) will be reported below. Initial tests with make-up solvent flow rates of 6:4 (0.45 mL/min) or higher generally resulted in smaller peak areas and broader peaks. Likewise, make-up flow rates of <0.08 mL/min resulted in higher relative standard deviations (RSDs), probably due to a more variable admixture rate at low flow rates. These results will not be shown below for reasons of clarity.

### Investigations of matrix effects

Standard solutions containing the analytes at the same concentration, prepared in ultrapure water or blank matrix extract, were alternately injected into the AS19 column. Matrix effects were calculated according to Eq. 1 [28]:

1 $$ME \left[\%\right]= \left(\frac{B}{A}-1\right)\cdot 100$$

with: ME = matrix effect in %; with “0” meaning no matrix effect and “−100%” meaning total suppression, A = signal intensity/peak area of the analyte in solvent (here: water), and B = signal intensity/peak area of the analyte in matrix extract

Matrix effect experiments were performed with four problematic matrices whose extracts were known to be rich in specific anionic or potentially anionic components, i.e., lemon (~45 mg/g citric acid [29]), soybean (~5.5 mg/g phosphate [29]), rhubarb (~3 mg/g oxalic acid and ~12 mg/g malic acid [29]), and Swiss chard (~5 mg/g nitrate [29]). Cucumber, which is known for providing extracts with a low load of matrix components, was included for comparison [29]. The contents of some exemplary matrix components with anionic or potentially anionic character in the abovementioned matrices and their expected concentration in the raw QuPPe extracts are shown in Table S5 (supplementary material) [29]. With the exception of soybeans (5 g milled material), 10 g cryogenically milled homogenate was extracted according to the QuPPe method, and the blank extracts were spiked at 0.05 µg/mL with nine analytes (glyphosate, aminomethylphosphonic acid (AMPA), *N*-acetyl-glyphosate (NAGly), fosetyl, ethephon, 2-hydroxyethylphosphonic acid (HEPA), glufosinate, 3-methyl-phosphinicopropionic acid (MPPA), *N*-acetyl-glufosinate (NAGlu)). IL-ISs of each compound were added at the same concentration but most data will be presented without IL-IS corrections in order to illustrate matrix effects (IL-ISs were only included to verify the *t*_R_ of glyphosate, see below). QuPPe extracts were measured and evaluated directly, as well as following a fivefold, 10-fold, and 20-fold dilution with ultrapure water, respectively. Signal suppressions/enhancements due to matrix effects, *t*_R_ shifts, and the absolute signal intensities were studied and compared to find the optimum between dilution rate and matrix effect compensation.

During method validation, matrix effects in IC- and LC-MS/MS were studied for nine of the 14 compounds included in this study (glyphosate, AMPA, NAGly, glufosinate, MPPA, NAGlu, fosetyl, ethephon, HEPA) in seven matrices (cucumber, strawberry, rice, soybean, milk, liver, kidney). These experiments involved IC-MS/MS with the AS19 column and LC-MS/MS with either PGC or HILIC (Torus DEA) columns. In detail, the chromatographic peaks obtained from a solvent-based standard were compared to those of standards added to the blank matrix extracts (see above) and the extent of the matrix effect was calculated via Eq. 1 (see above). The spiking levels of AMPA and glyphosate in this experiment were 0.1 mg/kg (cucumber, strawberry, milk, liver, kidney) or 0.2 mg/kg (soybean and rice), while glufosinate was spiked at 0.06 mg/kg (cucumber, strawberry, milk, liver, kidney) or 0.12 mg/kg (soybean and rice). Other compounds were also evaluated for matrix effects, but results will not be shown in this manuscript. IL-ISs were added to the calibration standard after extraction in the respective concentrations (Table S2) for calculation of recovery rates (see below). For IC-MS/MS and LC-MS/MS using the PGC column, all extracts and matrix-matched calibration standards were diluted fivefold before injection, with the exception of strawberry extracts when using the PGC column. Using the HILIC column, all extracts were diluted fivefold before injection.

### Measurement of matrix effect profiles

Matrix effect profiles were measured to visualize signal suppressions and enhancements occurring during IC-MS/MS and LC-MS/MS runs. Specifically, blank matrix extracts (here: lemon, soybean) were injected while continuously introducing a mixture of the standard substances (glyphosate, AMPA, NAGly, fosetyl, ethephon, HEPA, glufosinate, MPPA, NAGlu, cyanuric acid, chlorate, perchlorate, and phosphonic acid; 0.5 µg/mL each) via a T-piece in front of the ion source (a.k.a. post-column infusion). Matrix effects were determined relative to peak areas of the ion chromatograms of MS/MS transitions (Table S4) in runs with pure solvent composition (no matrix) using Eq. 1 and for each data point plotted against *t*_R_. Extreme points in the resulting matrix effect profiles indicated *t*_R_ segments particularly prone to matrix effects. The calculated matrix effect at each measurement point was displayed as a curve (abundance over *t*_R_), which was overlaid with conductivity chromatograms (in the case of IC) or total ion chromatograms (TICs, in the case of LC-MS/MS) to visualize the inverse correlation between the two displays. Profiles were generated for undiluted extracts (LC-MS/MS, HILIC column) and fivefold diluted extracts (IC-MS/MS). In IC runs, conductivity was measured just after the chromatographic column and the suppressor, while MS data were recorded after the eluate passing the distance between the two instruments through a capillary. To account for the resulting *t*_R_ gap between both measurements, the profiles were shifted by ~0.2 min relatively to the conductivity chromatograms. The TICs in LC-MS/MS using HILIC were generated by injecting a blank matrix extract without post-column infusion. In this case, the full scan chromatogram (*m/z* 50–*m/z* 1250) was recorded in the first quadrupole (Q1).

### Method validation

Method validation was performed with matrices of plant and animal origin. Since fosetyl IL-IS is often contaminated with native phosphonic acid [16], the analytes were divided into two groups in this step. Analytes of group 1 (glyphosate, AMPA, NAGly, ethephon, fosetyl, HEPA, glufosinate, MPPA, NAGlu, cyanuric acid) were validated in red currants, cucumber, liver, rice, soybean, strawberry, and milk following the protocol of an interlaboratory validation study (liver, rice, soybean, strawberry, milk) organized by the EURL-SRM and additionally at a lower level in red currants, milk and cucumber. Analytes of group 2 (chlorate, perchlorate, bromide, phosphonic acid, TFA) were validated in milk, lemon, pumpkin puree, and sesame. The samples were spiked with the native analytes and the corresponding IL-IS followed by the conduction of the QuPPe method [16]. Spiking levels were adapted to the respective matrix and analyte (see below). Average recovery rates achieved in five replicate experiments were calculated using a 2-point matrix-matched bracketing calibration at 60% and 120% of the spiking level. Additionally, either cucumber-based (in the case of rice, soybean, liver, milk in Table 3) or solvent-based (in the case of red currants, cucumber, lemon, pumpkin puree, sesame, milk in Table 4) calibration was conducted. IL-ISs were used to compensate for matrix effects and other sources of bias (including low recovery rates) and added between 0.1 and 0.4 mg/kg on 10 g sample portion (Table S2). The performance followed the criteria of the European guidance document SANTE/12682/2019 [30]. Extracts of an interlaboratory validation study were measured both by IC-MS/MS (AS19 column) and LC-MS/MS (HILIC and PGC columns).

## Results and discussion

### Ion chromatography — optimization on the AS19 column

**Fig. 1:**
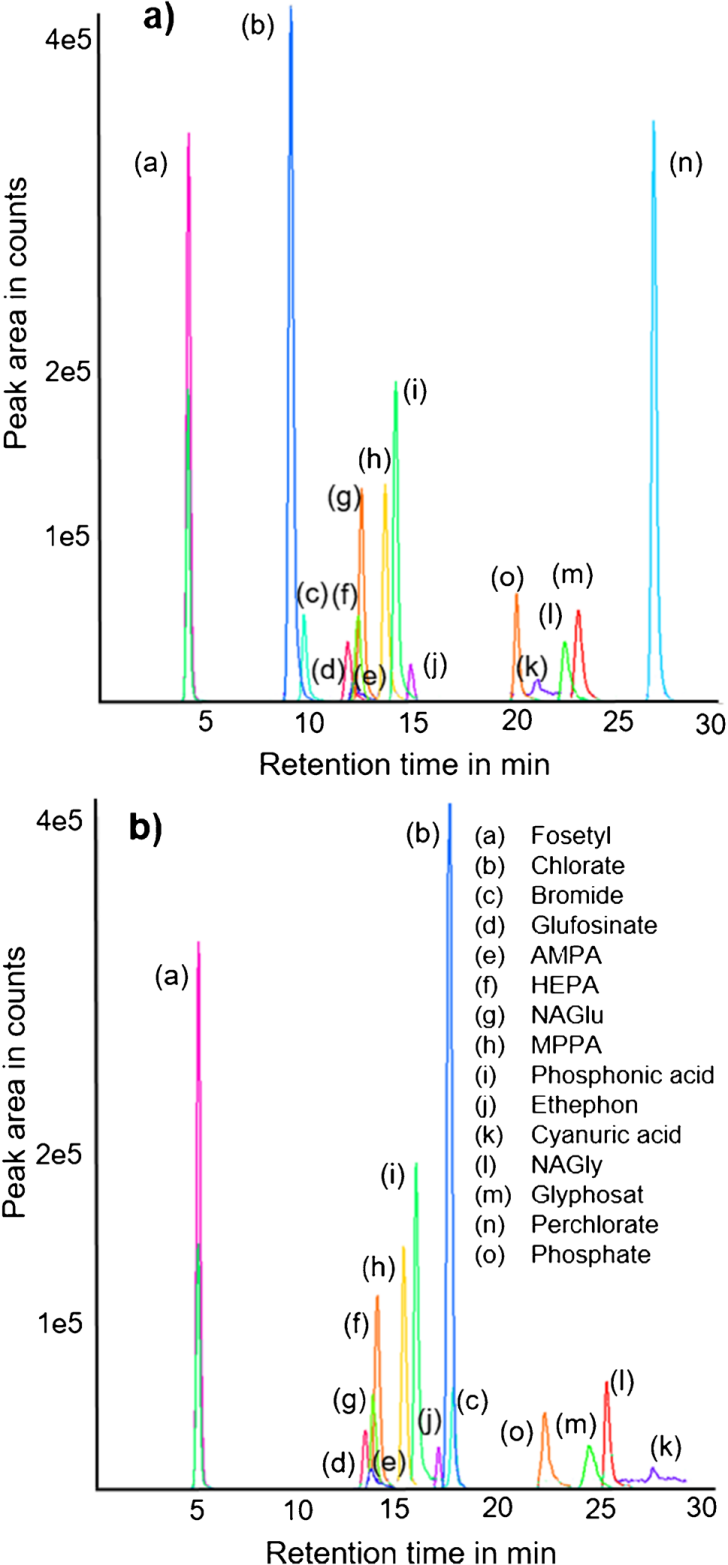
IC-MS/MS chromatograms of 14 analytes (peaks #a-#n) and the matrix compound phosphate (peak #o) obtained on (**a**) the AS19 column and (**b**) the AS24 column, each compound at 0.01 µg/mL in water. Note that the peak of perchlorate (#n) is not displayed in (**b**) due to its *t*_R_ > 40 min on the AS24 column

Initial tests with four anion separation columns (AS11, AS18, A19, and AS24) indicated that the AS19 and the AS24 columns performed the best (details not shown). Initially, also an IC phase designed for for bicarbonate elution was tested. However, performance was poor in the used IC system, which is technically specialized on hydroxide elution. The main advantage of the AS19 column (Fig 1a) was the weaker retention of polarizable analytes, mainly perchlorate (Fig 1a, b, #n), which eluted more than 10 min after the penultimate analyte from AS24 (Fig. 1b). Even at OH^–^ concentrations of >60 mM, the *t*_R_ of perchlorate on AS24 was >40 min. In contrast, peaks in AS24 chromatograms were narrower and less tailing than in AS19 chromatograms. However, due to the shorter run time (<30 min), the AS19 column was deemed more beneficial for high-throughput routine operation than the AS24 column. Yet, if perchlorate (and similarly late eluting anions) is not to be analyzed, it is recommended to use the AS24 column. Subsequently, different conditions on the AS19 column were tested with the goal of a good separation of the 14 analytes within 30 min.

Specifically, the concentration of hydroxide ions (OH^-^) in the eluent was varied to separate critical pairs. Emphasis was put on the separation of glyphosate from its metabolite NAGly, which generates ions used for the determination of glyphosate in the ESI ion source. A sufficient resolution (*R*) >1 of glyphosate and NAGly was achieved at an OH^–^ concentration of 36 mM, while *R* sank below 1 at OH^–^ concentrations < 32 mM and > 42 mM, leveling out at *R* ≈ 0.2 at OH^–^ concentrations < 25 mM or > 60 mM.

Substances that are sometimes difficult to separate by HPLC methods (LC-MS/MS) can be resolved effortlessly by IC. Specifically, fosetyl (singly charged at alkaline conditions) was well-separated from both phosphonate (doubly charged) and phosphate (triply charged) (Fig. 1a, peaks #a, #i, #o) by IC-MS/MS, while this is difficult to achieve by LC-MS/MS when PGC is used as stationary phase (Fig. S2, supplementary material). However, the separation of these three components is crucial for quantitative studies since both fosetyl and phosphate interfere with phosphonate due to fragmentation in the ESI source [16]. Phosphate, which naturally occurs at high concentrations in many food products, shares the most abundant MS/MS transition (*m/z* 81→63) with phosphonate. Only the less abundant MS/MS transition *m/z* 81→79 of phosphonate is largely unaffected by phosphate and thus may be used for quantification. To obtain the requirements for identification [30], it is essential to separate phosphonate chromatographically from phosphate. In this context, it is important to note that commercial standard solutions of fosetyl (and fosetyl-D_5_, Table S2) are often contaminated with native phosphonic acid, originating both from impurities in the neat standards and from the hydrolytic degradation of fosetyl in solutions [16].

### Effect of the make-up solvent (type and flow rate) on the peak area in ESI-MS with particular attention on glyphosate and AMPA

Contrary to LC-MS/MS effluents, which consist of mixtures of water and a water-miscible organic solvent, the IC effluent is purely aqueous. Yet, the composition of the effluent has an influence on its evaporability, the surface tension, the stability of ion spray droplets, and ultimately the ion yield in an ESI source [31, 32]. In this context, pure aqueous solutions are not ideal for achieving high ion yields in ESI mode [32]. This disadvantage can be overcome by post-column infusion of organic solvents [20, 23]. For this purpose, methanol, ACN, or 2-propanol was individually evaluated at five make-up/IC flow rate ratios of ~1:4 to ~5:4 relative to the constant IC (solvent) flow rate 0.3 mL/min (see “Experimental”). Particular attention was put on the performance of glyphosate and AMPA, because these analytes provided poor detector responses (either generally or as a result of matrix effects). In general, the RSD of the triplicate analyses was small (typically ~5%; scarcely >10%, Table 1 and Table S6), and therefore, the following discussion could be based on mean values.

**Table 1:** Exemplary effect of the make-up solvents ACN, methanol, and 2-propanol at ratios of ~1:4 to ~5:4 compared to a constant IC flow rate of 0.3 mL/min for glyphosate, AMPA, and NAGly. The % value of the peak areas was compared with injections in pure water (no make-up solvent), which was set at 100%. Each measurement was performed in triplicate and mean values (and relative standard deviation (RSD) in parentheses) were given for the most prominent MS/MS transitions. The best values are highlighted in bold letters. Corresponding data of 12 compounds is shown in the supplementary material (Table S6)

Compound	Solvent	Flow rate of make-up solvent (external pump)*				
		0.08 mL	0.15 mL	0.23 mL	0.3 mL	0.38 mL
		Make-up solvent flow rate compared to IC effluent flow rate (1:1 = equal)				
		~1:4	2:4 (1:2)	~3:4	4:4 (1:1)	~5:4
		Share of make-up solvent on total flow after admixture				
		20%	33%	43%	50%	56%
		Normalized peak areas (no make-up solvent set at 100%); RSD in % in brackets				
Glyphosate	Acetonitrile	**189%** (7%)	**180%** (6%)	**197%** (5%)	175% (1%)	162% (3%)
	Methanol	**153%** (5%)	123% (5%)	103% (2%)	86% (7%)	71% (7%)
	2-Propanol	100% (19%)	**150%** (10%)	86% (1%)	87% (3%)	79% (4%)
AMPA	Acetonitrile	**196%** (4%)	168% (3%)	**201%** (4%)	**186%** (5%)	175% (6%)
	Methanol	**144%** (4%)	117% (4%)	107% (5%)	86% (1%)	65% (15%)
	2-Propanol	105% (13%)	**131%** (5%)	98% (14%)	88% (9%)	100% (7%)
NAGly	Acetonitrile	**223%** (5%)	169% (3%)	181% (12%)	138% (2%)	146% (7%)
	Methanol	**236%** (7%)	**232%** (3%)	**248%** (9%)	201% (12%)	152% (11%)
	2-Propanol	112% (20%)	**182%** (11%)	124% (10%)	136% (6%)	125% (8%)

^*^The flow rate of AXP-MS pump was only adjustable to two decimals. Therefore, increments derived from the standard value of 0.3 mL (i.e., identical with the flow rate of the mobile phase) were rounded to two decimals

Using ACN at make-up/IC flow rate ratios of 1:4 to 3:4 almost doubled the peak areas of glyphosate compared to the reference value (=100%) without the use of a make-up solvent (189%, 180%, and 197% respectively, Table 1). However, higher make-up/IC flow rate ratios (4:4, 5:4) resulted in slight drops of the signal by ~10–15% (Table 1) (see also Fig. S3 in the supplementary material). With methanol, the highest increase in the peak area of glyphosate (~150% of the reference value) was already obtained at the lowest make-up/IC flow rate ratio of ~1:4. An increase of the methanol flow rate led to a continuous drop of the peak area of glyphosate, which fell below the reference value at a make-up/IC flow rate ratio of 4:4 (1:1) or ~5:4 (Table 1). With 2-propanol as make-up solvent, peak area of glyphosate could only be increased at a 2:4 (1:2) ratio of the make-up/IC flow rate (150%, Table 1), and this solvent was the least suitable as make-up solvent with the present instrument and setup.

With a few exceptions, the benefit of using make-up solvents on the peak area of AMPA dropped in the order ACN > methanol > 2-propanol irrespective of the make-up/IC flow rate ratio (Table 1). Also, moderate make-up solvent flow rates, i.e., those lower than the IC flow rate, resulted in the best performance for AMPA (Table 1).

Several other pesticides showed similar trends as glyphosate and AMPA, but to different extents. With the exception of NAGly, NAGlu, and chlorate (highest peak areas with methanol, Table 1 and Table S6), ACN induced the highest increase in the peak area of all analytes. The extent was largest for perchlorate, where a ~1:4 ratio of ACN/IC flow rate resulted in a more than fourfold increase of the peak area relative to the pure aqueous solution. Overall, ACN turned out to be best suited to improve the peak areas of the present analytes. In addition, ACN has also the lowest viscosity (pure and in mixtures with water) of the three solvents, which was favorable as its application omitted the build-up of high back pressures and thus had a protective effect on the suppressor [20, 33]. Accordingly, subsequent experiments were carried out with ACN as the make-up solvent for the polar pesticides and analytes tested in this study.

Considering all aspects, a flow rate of 0.15 mL/min ACN admixed to the IC flow rate of 0.3 mL/min (make-up/IC flow rate ratio of 2:4 (1:2)) represented the best compromise and was subsequently used in all further experiments. This ACN make-up/IC flow rate ratio of 2:4 (1:2) was located between the ratio of 1:1 (Rajski et al. [23]) and 1:2.75 (Adams et al. [20]) previously used for the determination of polar pesticides. This could be due to different IC settings or different geometries of the ESI source. Accordingly, different ACN make-up/IC flow rate ratios should be tested when a method is transferred to other settings or instruments.

### Investigation and management of matrix effects during the IC-MS/MS determination of highly polar pesticides

QuPPe extracts of highly polar pesticides obtained from food regularly contain co-extracted matrix components of similar polarity, such as soluble carbohydrates, soluble proteins/peptides, organic acids, free amino acids, and also inorganic anions, such as nitrate and phosphate [17–19]. These are barely removed by subsequent clean-up steps of the QuPPe extraction method (i.e., freezing-out, dilution with ACN, particle filtration, and SPE with C_18_ sorbent [16]). In the case of co-elutions, these co-extracted compounds may cause (i) *t*_R_ shifts of analytes, (ii) signal suppression in the (ESI) ion source, and (iii) interferences in (MS and) MS/MS chromatograms [17, 31]. The relevance of these effects can vary strongly from matrix to matrix and thus needs to be examined for each matrix type to circumvent quantification errors. Practically, undesired matrix effects can be reduced either by the dilution of sample solutions or by the performance of additional clean-up steps [34]. Also, matrix-matched calibrations and IL-ISs are frequently used to compensate for matrix effects [30]. Typical matrix compounds in food that may affect IC- and LC-MS/MS analysis of anions are organic acids (e.g., citric, malic, and oxalic acids) and inorganic anions (e.g., phosphate, chloride, and nitrate) [17, 18].

In this study, matrix effects were determined by spiking the same amount of standards in water and the (pesticide-free) blank extract (see “Experimental” section), followed by evaluation by means of Eq. 1. Variations of matrix effects observed in replicate injections were small in the case of strong matrix effects, but larger when matrix effects were weak, which was however of minor relevance in practice. For the sake of simplicity, the subsequent results are presented without reporting the (relative) standard deviations. Also, matrix effects were examined without IL-IS correction. It was only evaluated whether the respective IL-IS was able to correct for the observed effects.

- Matrix-induced signal suppressions and retention time (*t*_R_) shifts

**Fig. 2:**
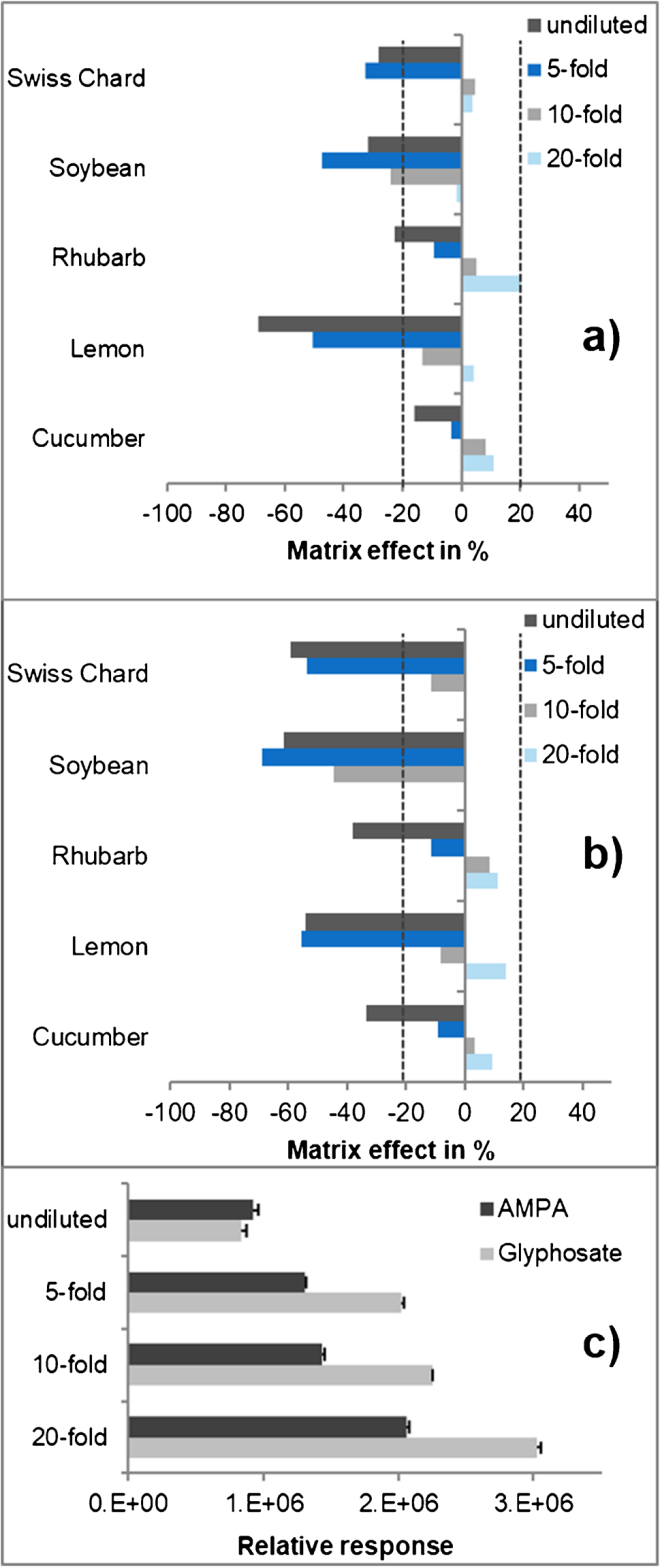
Extent of matrix effects of (**a**) glyphosate and (**b**) AMPA in undiluted and fivefold, 10-fold, and 20-fold diluted QuPPe extracts of lemon, soybean, Swiss chard, rhubarb, and cucumber. (**c**) The relative responses of the peak area of glyphosate and AMPA in lemon extracts undiluted and following fivefold, 10-fold, and 20-fold dilution with ultrapure water (*n*=3). No result for ME of AMPA in 20-fold dilution of soybean and Swiss chard is shown due to too poor signals

*Signal suppressions.* Five QuPPe extracts (cucumber, lemon, rhubarb, soybean, and Swiss chard) were examined for matrix effects of glyphosate and AMPA in dependence of four matrix concentrations (undiluted, 5-, 10-, and 20-fold diluted). Signal suppression/enhancements of glyphosate and AMPA were observed in all matrices, but the extent was dependent both on the matrix type and the dilution factor of the solutions (Fig. 2a, b). Specifically, the relative response increased with increasing dilution, albeit to different extents (Fig. 2c).

*Matrix effects in undiluted QuPPe extracts*. Usually, matrix effects between −20 and 20% are considered negligible (dotted lines in Fig. 2a, b) [30]. In all cases, signal suppression was observed and, with the exception of glyphosate in cucumber, the margin of −20% was exceeded by glyphosate and AMPA (Fig. 2a, b). For AMPA, signal suppression was severe (>−50%) in soybean, lemon, and Swiss chard (Fig. 2b). The strongest signal suppression for glyphosate was also observed in lemon extract (Fig. 2a). This was mainly due to the similar *t*_R_ of glyphosate and citrate, which not only induced signal suppressions but also *t*_R_ shifts (see below).

Fortunately, the abundant phosphate did not co-elute with any of the analytes under the applied IC conditions. However, high amounts of nitrate such as in Swiss chard resulted in a broad peak, which partly co-eluted with AMPA (and also glufosinate, chlorate, and bromide). Nitrate was therefore supposed to be responsible for the strong signal suppression of AMPA in the Swiss chard extract (Fig. 2b). However, the similarly strong signal suppression (>−60%) of AMPA in lemon and soybean extracts (Fig. 2b) could not be traced back to nitrate or any other of the studied matrix components. In the following, the QuPPe extracts were diluted in three steps and evaluated.

*Matrix effects in 5-, 10-, and 20-fold diluted QuPPe extracts*. Matrix effects of glyphosate and AMPA fell within the acceptable range of ±20% even at 10-fold dilution, except for soybean (Fig. 2a, b). However, strong dilutions also decreased the peak areas of the analytes, so that they were too small to be evaluated, as in the case of AMPA in Swiss chard and soybean (Fig. 2b). Low-intensity signals are usually accompanied by higher standard deviations and this could also be the reason for the slight signal enhancement effects observed at 20-fold and partly also at 10-fold dilutions (Fig. 2). Fortunately, IL-IS could be used to correct for such matrix effects, even at matrix effects of more than ±20%. Specifically, IL-IS-corrected recovery rates ranged between 90 and 120%, regardless of the dilution factor (if the pesticide was detectable). Overall, 20-fold dilutions of QuPPe extracts were deemed less favorable than 5- or 10-fold dilutions, but they may still be useful in cases where a second analysis is needed due to high residue levels. To make this possible, spike levels of IL-IS were chosen to achieve *S*/*N* > 100 at 20-fold dilution.

**Fig. 3:**
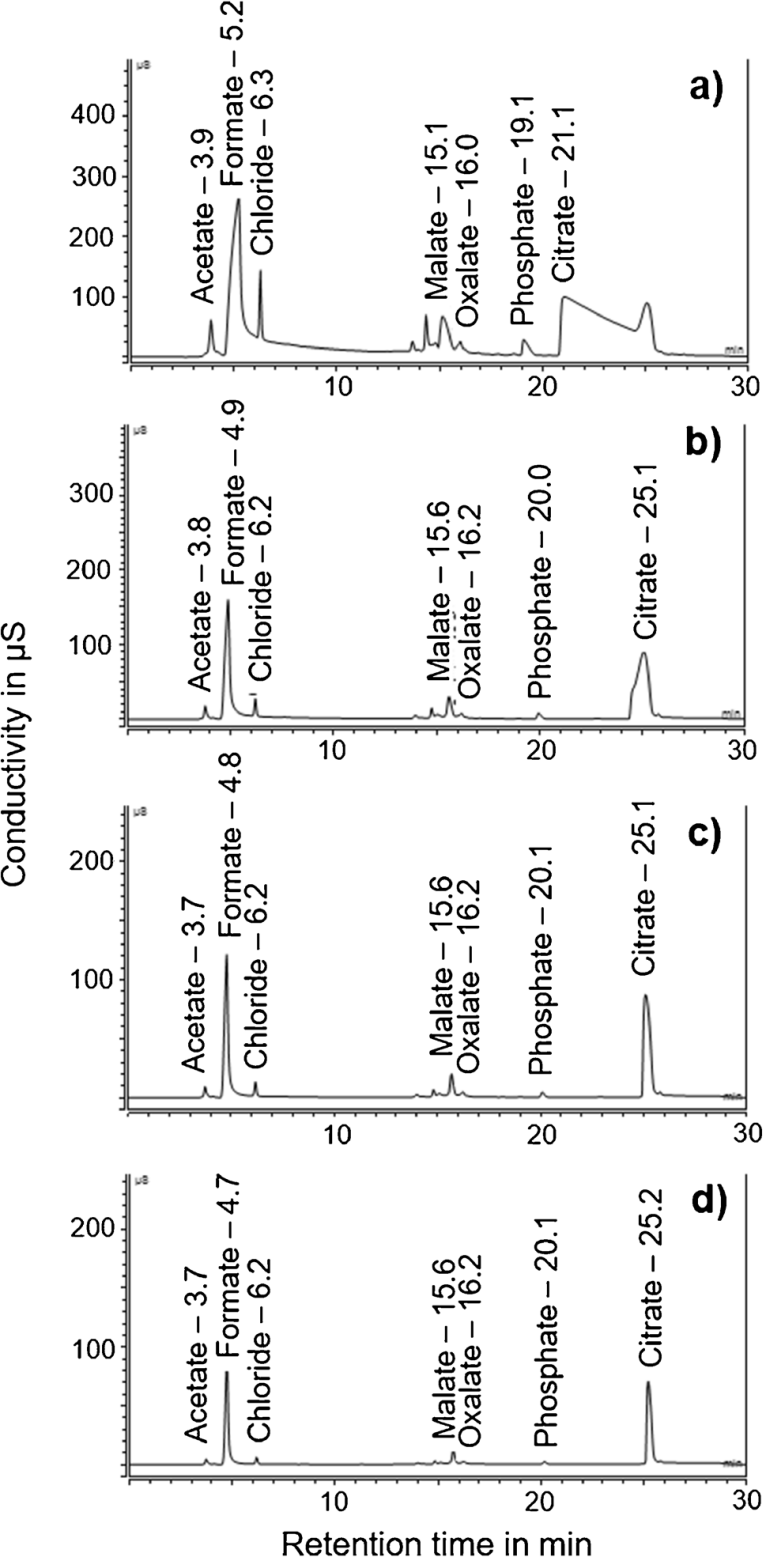
IC conductivity chromatograms (AS19 column) of lemon extracts undiluted (**a**), following fivefold dilution (**b**), 10-fold dilution (**c**), and 20-fold dilution (**d**) showing some known anionic matrix components. Values behind the analytes are IC retention times

*Retention time (t*_*R*_*) shifts*. High levels of certain anionic matrix components in QuPPe extracts may lead to an overload of IC columns. While some small inorganic anions like chloride eluted as narrow peaks even at high concentrations, this was not the case for other matrix components. For instance, high amounts of citrate in lemon extracts and formate from the QuPPe solvent caused broad bands in the undiluted sample extract (Fig. 3). The resulting peak broadening of the matrix component not only increases the risk of co-elutions with analytes, but may also cause shorter *t*_R_ of affected analytes due to the competition with interaction sites. Specifically, overload by anionic matrix components (here: citrate) decreased the *t*_R_ of glyphosate by >2 min in the undiluted lemon extract (Fig. S4 in supplementary material). As a consequence, glyphosate partly co-eluted with NAGly, which must be avoided since NAGly may be transformed into glyphosate in the ESI ion source (which will falsify the result of the latter). However, this problem only existed in undiluted lemon QuPPe extracts (Fig. S4 in supplementary material). Already a fivefold dilution of the samples virtually eliminated *t*_R_ shifts and all issues caused by them in the undiluted lemon extract (Fig. 3a, b). Specifically, the broad band between 20 and 25 min was narrowed and *t*_R_ shifts of analytes were no longer a problem. This positive effect of sample dilution could be verified by conductivity measurements. The signal of the citrate peak area dropped roughly proportionally from 302 µS*min (undiluted extract) to 64, 31, and 17 µS*min in the fivefold, 10-fold, and 20-fold diluted extracts, respectively.

Further matrix component peaks (indicated by broader peaks in undiluted QuPPe extracts) were observed for phosphate and various doubly charged organic matrix compounds (such as malate) and formate (which originated from the extraction solvent). Again, all of these matrix compounds eluted as sharp peaks after a fivefold dilution. Overall, it was concluded that, based on a sample weight of 10 g and an extraction volume of 20 mL, a fivefold dilution of QuPPe extracts was a good compromise for the determination of highly polar pesticides and related compounds. On the one side, this measure removed or minimized critical matrix effects and also improved the lifespan of columns by omitting permanent overloads. On the other hand, it still enabled to determine specific analytes that were close to LOD in a second run with an aliquot of the undiluted QuPPe sample extract. As the QuPPe method was designed as a multimethod for a range of polar analytes and as QuPPe extracts are also injected undiluted in other applications (such as for analytes that are measured by LC-MS/MS in the ESI positive mode) [16], an initial reduction of the sample weight by a factor of 5 was considered more restrictive and thus less suitable (also since this approach may affect the precision of the analysis in case of inhomogeneity of the sample material).

While every dilution step decreased Δ*t*_R_ between glyphosate and NAGly in the sample and standard, the deviation from the *t*_R_ in pure water (no matrix, which is the basis of calibration standards) was still >0.1 min, which is the threshold stipulated in the SANTE quality control and method validation procedures document, where no IL-IS is used [30]. Strong *t*_R_ shifts, as observed with glyphosate and NAGly in the undiluted (lemon) QuPPe extract, pose the risk that analytes are overlooked. This in turn increases the risk of false negative results, especially if no IL-IS is used, whose absence from the screened *t*_R_ window would trigger further investigations to avoid a false negative result. In such a context, the use of IL-IS plays a decisive role in quality control. In agreement with expectations, the *t*_R_ of the native substances matched with those of the respective IL-IS (*t*_R_ ratio = 1.00) in both the undiluted and diluted (fivefold, 10-fold, and 20-fold) state of the QuPPe extract. Accordingly, *t*_R_ shifts and/or an abnormal peak shape of the IL-IS and/or a significant suppression of the IL-IS would be a direct indication of matrix effects. However, apart from effects of citrate on glyphosate in QuPPe extracts of lemon, severe *t*_R_ shifts of the 14 analytes were negligible in the other five matrices studied.

*Matrix effects of glyphosate, glufosinate,* *and AMPA in IC-MS/MS versus LC-MS/MS using seven matrices (cucumber, strawberry, soybean, rice, milk, liver, kidney).* In IC-MS/MS, glyphosate performed well in all tested matrices and all values in the fivefold diluted QuPPe extracts of the seven matrices were within ±20% deviation (except for soybean with −22%) (Table 2). With one exception (glyphosate, IC-MS/MS, +5%), the observed matrix effects were caused by signal suppression. In contrast, using LC-MS/MS, all but one (LC-MS/MS (PGC)) or two values (LC-MS/MS (HILIC, undiluted extracts, data not shown)) of glyphosate deviated more than 20%. However, when injecting fivefold diluted extracts on HILIC, all but two values were within the acceptable range (Table 2).

**Table 2:** Matrix effects observed for AMPA, glyphosate, and glufosinate during QuPPe method validation of seven exemplary matrices with three different methods, i.e., IC-MS/MS (AS19), LC-MS/MS (HILIC), and LC-MS/MS (PGC). All extracts were fivefold diluted, except for strawberry with PGC (undiluted)

Analyte	Method	Cucumber	Strawberry	Soybean	Rice	Milk	Liver	Kidney
AMPA	IC	−39	−23	−46	−49	−33	−33	−53
	HILIC	−76	−70	−91	−13	−66	−79	−58
	PGC	−93	−86*	−53	−7	−10	−83	−87
Glyphosate	IC	18	−7	−22	−7	5	−10	14
	HILIC	1	−37	−23	19	−9	−4	9
	PGC	−39	−58*	25	−34	−5	−42	58
Glufosinate	IC	−46	−27	−48	−45	−30	−13	−42
	HILIC	−87	−84	−57	0	−31	−47	−28
	PGC	−80	−76*	−39	7	−32	−70	−56

^*^PGC with undiluted strawberry extracts

AMPA and glufosinate performed worse than glyphosate in IC-MS/MS (deviations between −13 and −53%), but still better than in most LC-MS/MS measurements. For instance, of the 21 measurements (three pesticides in seven matrices), IC-MS/MS performed best 12 times and only 2 times worst (i.e., AMPA and glufosinate in rice, Table 2). Overall, however, deviations by less than ±20% from the target value (compared to solvent) were almost as frequent in IC-MS/MS as with LC-MS/MS using the HILIC column (7 times vs. 7 times with HILIC and 3 times with PGC). Yet, deviations in IC-MS/MS were more consistent, that is, less matrix-dependent. For instance, deviations of AMPA in the seven matrices varied only by 30% (−23 to −53%) in IC-MS/MS compared to 78% (−13 to −91%) in LC-MS/MS (HILIC) and 86% (−7 to −93%) in LC-MS/MS (PGC) (Table 2). Accordingly, the effect was overall more predictable (and manageable) in IC-MS/MS.

This good performance of the IC-MS/MS method could be solidified by considering all 63 matrix compound measurements (nine compounds in seven matrices). Specifically, IC-MS/MS showed negligible matrix effects in 40 cases (63%). This quote was between the one in LC-MS/MS (HILIC) with 42 cases (67%) and LC-MS/MS (PGC) with 31 cases (45%). Overall, the average signal suppression was −14% in IC-MS/MS, −18% in LC-MS/MS (HILIC), and −23% in LC-MS/MS (PGC). Overall, LC-MS/MS (HILIC) and IC-MS/MS (AS19) were less affected by matrix effects than LC-MS/MS (PGC), with the IC-MS/MS method performing slightly worse than LC-MS/MS (HILIC) method in terms of matrix effects. However, the observed matrix effects in IC-MS/MS were more consistent, and thus better manageable. The main benefit of the IC-MS/MS method was its better performance for the highly relevant glyphosate, AMPA, and glufosinate.

(b) Comparison of conductivity chromatograms and post-column infusion matrix effect profiles

**Fig. 4:**
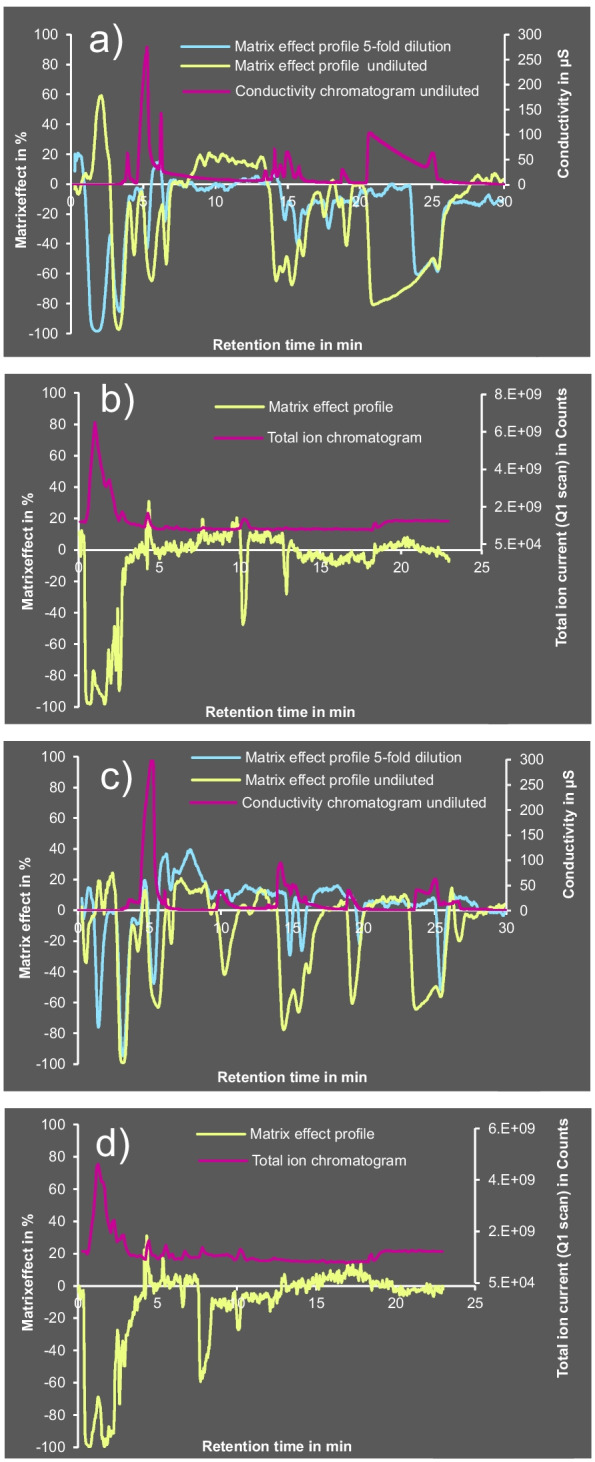
Matrix effect profiles (MEPs) of undiluted lemon extract (**a** (IC) and **b** (LC-HILIC), yellow) and undiluted soybean extract (**c** (IC) and **d** (LC-HILIC), yellow) along with the corresponding fivefold dilutions (**a**, **c** (IC), pale blue) overlaid by IC conductivity chromatograms (**a**, **c** purple) and LC-MS/MS TICs (**b**, **d** purple); all extracts in LC-MS/MS were undiluted. TIC, total ion chromatogram

Continuous post-column infusions of a mix with glyphosate, AMPA, NAGly, and 10 further substances into blank matrix extracts (see “Experimental”, Matrix effect profiles) were used to visualize matrix effects in both IC- and LC-MS/MS in form of negative peaks (Fig. 4).

Several signal suppressions of analytes could be largely eliminated at a fivefold dilution (see *t*_R_ ranges at ~10 and ~20 min in the IC-MS/MS chromatogram of the soybean extract (Fig. 4c) and at ~20–23 min in the lemon extract (Fig. 4a; yellow vs. blue profile)). Signal suppressions at short IC-MS/MS *t*_R_ (<4 min) originated from non-ionic or weakly ionic matrix compounds such as terpenes, sugars, or peptides (Fig. 4a, c). Since all ionic analytes except fosetyl (~4.2 min) eluted after 8 min (Fig. 1a), these compound classes posed no problem in IC-MS/MS runs. In LC-MS/MS using HILIC, very strong signal suppressions (with ~–70 to –100% matrix effect) also occurred within the first ~4 min (Fig. 4b, d). Contrary to the IC-MS/MS method, however, several important analytes (AMPA, glyphosate, MPPA, glufosinate, NAGlu [16]) eluted in this range (i.e., before 3 min) from the LC-MS/MS column, which agreed well with the partly substantial signal suppressions observed for these analytes (Table 2). Consequently, conductivity chromatograms in IC and TICs in LC-MS/MS proved to be valuable tools for roughly assessing the occurrence and intensity of matrix effects during routine analysis.

### IC-MS/MS method validation results and comparison to LC-MS/MS

Following the method developments shown above, the finally applied IC-MS/MS method used an AS19 column, a mobile phase flow of 0.3 mL/min, the make-up solvent ACN with a flow rate of 0.15 mL/min, and fivefold diluted QuPPe extracts. After IL-IS correction, the recovery rates of all spiked pesticides met the requirements regarding recovery experiments in pesticide residue analysis (i.e., 80–120%, RSDs <20%) stipulated in the European guidance document SANTE/12682/2019 [30]. Specifically, ten analytes (including the “critical three” glyphosate, AMPA, and glufosinate) showed recovery rates between 86 and 116% (with only three values <90%) and RSDs between ~1 and 14% (except cyanuric acid in soybean and HEPA in bovine liver) (Table 3). Virtually, the same quality was obtained for five anions in four commodities (Table 4). Validation data using cucumber-based or solvent-based calibration showed overall comparable results to the matrix-matched approach, e.g., cucumber-based average recovery of AMPA in soybean was 102% (11% RSD), of glyphosate in soybean was 103% (3.9% RSD), of AMPA in liver was 89% (7.8% RSD), and of glyphosate in liver was 98% (2.7% RSD); and solvent-based average recovery of chlorate in lemon was 100% (0.4% RSD), of perchlorate in pumpkin puree was 106% (4.4% RSD), and of phosphonic acid in pumpkin puree was 100% (3.0% RSD). More validation data for IC-MS/MS and comparison to LC-MS/MS (using PGC and HILIC) measurements of strawberry and milk can be found within the supplementary material (Table S7).

**Table 3:** Average recovery rates of highly polar anionic pesticides (*n*=5) in red currants, cucumber, rice, soybean, milk, and liver using QuPPe extraction and IC-MS/MS on an AS19 column. QuPPe extracts were fivefold diluted prior to injection

Analyte	Red currants		Cucumber		Rice		Soybean		Milk		Liver	
	Level in mg/kg	Ave. recovery in % (±RSD in %)	Level in mg/kg	Ave. recovery in % (±RSD in %)	Level in mg/kg	Ave. recovery in % (±RSD in %)	Level in mg/kg	Ave. recovery in % (±RSD in %)	Level in mg/kg	Ave. recovery in % (±RSD in %)	Level in mg/kg	Ave. recovery in % (±RSD in %)
AMPA	0.01	96 (5.2)	0.02	110 (4.8)	0.1	96 (2.4)	0.1	105 (4.5)	0.01	115 (8.2)	0.05	96 (3.6)
Cyanuric acid	0.01	*	0.05	86 (14)	0.1	94 (10)	0.1	*	0.05	98 (12)	0.1	103 (12)
Ethephon	0.01	102 (7.9)	0.02	90 (5.2)	0.02	104 (14)	0.02	99 (10)	0.01	95 (3.6)	0.01	101 (10)
Fosetyl	0.01	104 (7.4)	0.02	101 (3.1)	0.02	89 (1.8)	0.02	102 (5.2)	0.01	100 (1.4)	0.01	103 (4.6)
Glufosinate	0.01	101 (1.8)	0.02	99 (4.6)	0.06	90 (2.3)	0.06	102 (1.8)	0.01	87 (5.3)	0.03	109 (6.2)
Glyphosate	0.01	101 (2.2)	0.02	96 (1.6)	0.1	116 (7.3)	0.1	105 (6.3)	0.01	98 (11)	0.05	100 (5.6)
HEPA	0.01	99 (0.9)	0.02	100 (4.9)	0.04	95 (6.8)	0.04	96 (8.3)	0.01	103 (8.8)	**	**
MPPA	0.01	102 (2.1)	0.02	103 (4.0)	0.04	95 (1.8)	0.04	104 (2.6)	0.01	97 (2.4)	0.02	100 (6.6)
NAGlu	0.01	101 (1.6)	0.02	103 (3.0)	0.04	96 (2.6)	0.04	98 (6.7)	0.01	108 (4.4)	0.02	102 (5.2)
NAGly	0.01	100 (0.9)	0.02	104 (2.7)	0.1	88 (14)	0.1	102 (4.0)	0.01	100 (13)	0.05	106 (3.7)

^*^Validation not successful at that level

^**^Validation not possible because of naturally occurring HEPA in bovine liver

**Table 4:** Average recovery rates of selected anionic polar compounds (*n*=5) in lemon, pumpkin (puree), sesame, and milk using QuPPe extraction and IC-MS/MS on an AS19 column. *TFA*, trifluoroacetic acid

Analyte	Lemon		Pumpkin puree		Sesame		Milk	
	Level in mg/kg	Ave. recovery in % (±RSD in %)	Level in mg/kg	Ave. recovery in % (±RSD in %)	Level in mg/kg	Ave. recovery in % (±RSD in %)	Level in mg/kg	Ave. recovery in % (±RSD in %)
Phosphonic acid	0.05	98 (0.4)	0.01	98 (3.0)	0.1	100 (3.0)	0.05	95 (2.2)
Chlorate	0.03	95 (2.7)	0.01	101 (1.1)	0.06	99 (5.2)	0.01	90 (9.1)
Perchlorate	0.01	99 (3.3)	0.01	100 (4.4)	0.02	114 (11)	0.01	102 (5.2)
Bromide	5	83 (10)	0.01	*	5	*	5	98 (3.8)
TFA	0.025	104 (12)	0.01	*	0.05	97 (9.1)	0.02	110 (5.7)

^*^Not evaluated due to high background levels

### Method performance

In the last step, 132 market samples with incurred residues were analyzed by IC-MS/MS (AS19) and LC-MS/MS (PGC). The sample set included fruits (*n*=58), vegetables (*n*=55), cereals (*n*=3), seven dried commodities (e.g., dried mushrooms, dried fruits), and nine other commodities (e.g., spices, smoothies, juice). Samples were measured with fivefold diluted QuPPe extracts using IC-MS/MS and LC-MS/MS (except for 10-fold dilutions for measurement of chlorate, perchlorate, phosphonic acid, and bromide). Main differences were observed for cyanuric acid (supplementary material Table S8), where IC-MS/MS indicated only eight positive samples (>LOQ) compared to 31 positive samples in LC-MS/MS (PGC). This was due to the 10 times higher LOQ in IC-MS/MS for this compound compared to LC-MS/MS. LC-MS/MS indicated more positive findings of fosetyl in the semi-quantitative concentration range at levels <LOQ, but with the analyte still being identifiable (4 times using LC-MS/MS with PGC compared to 2 times using IC-MS/MS, Table S8). In contrast, positive findings of glyphosate and HEPA within the semi-quantitative range were only found with IC-MS/MS (*n*=5 and *n*=6, respectively) but not in LC-MS/MS. This was due to the fact that both compounds were only marginally affected by matrix effects in IC-MS/MS. IC-MS/MS also performed better for phosphonic acid, chlorate, and perchlorate (more positive findings >LOQ) than LC-MS/MS (which provided higher LOQs, Table S8). Likewise, trifluoracetic acid (TFA), which could not be properly analyzed by LC-MS/MS (PGC) [16], was detected in almost 50% of all analyzed samples (31 samples >LOQ).

## Conclusion

The thorough investigation and evaluation of IC columns, make-up solvents, and their flows, as well as matrix effects, resulted in a valuable IC-MS/MS method for the analysis of highly polar pesticides and further analytes with similar properties. It could be demonstrated that the minimal sample clean-up of the QuPPe extracts was sufficient for IC-MS/MS (and mostly also LC-MS/MS) analysis when the resulting extracts were fivefold diluted before the instrumental analysis. The good recovery rates obtained for 14 analytes in various (problematic) matrices indicated that the method is well-suited for routine analyses, which was verified by the analyses of 132 samples arbitrarily collected from the market at various places.

### Supplementary Information

Below is the link to the electronic supplementary material.

Supplementary file1 (PDF 1014 KB)

## Declarations

### Source of biological material

Biological material from organic production (of plant and animal origin) was purchased at local supermarkets.
