# Supplementary material for: Analysis of highly polar anionic pesticides in food of plant and animal origin by ion chromatography and tandem mass spectrometry with emphasis on addressing adverse effects caused by matrix co-extractives
Source: Anal Bioanal Chem. 2024 Jun 19;416(20):4503–17. doi: 10.1007/s00216-024-05389-4 (PMC11294259; doi:10.1007/s00216-024-05389-4)
Supplement: Supplementary file 1 — Supplementary file1 (PDF 1014 KB) [file 216_2024_5389_MOESM1_ESM.pdf]

Supplementary Information for

Analysis of Highly Polar Anionic Pesticides in Food of Plant and Animal Origin by Ion Chromatography and Tandem-Mass Spectrometry with emphasis on addressing adverse Effects caused by Matrix Co-extractives

in *Analytical and Bioanalytical Chemistry*

Ann-Kathrin Schäfer[1]*, Walter Vetter[2] and Michelangelo Anastassiades[1]

[1] Chemisches und Veterinäruntersuchungsamt Stuttgart, Section of Residues and Contaminants, Fellbach, DE 70736, Germany

[2] University of Hohenheim, Institut für Lebensmittelchemie, Garbenstr. 28, Stuttgart, DE 70593, Germany

*corresponding author

Email address: Ann-Kathrin.Schaefer@cvuas.bwl.de

Table S1: *Brief comparison of the presented method in this manuscript to other IC-MS/MS methods [1-5].*

	**Method presented here**	**Adams et al. [1]**	**Raijski et al. [2]**	**Bauer et al. I [3]**	**Bauer et al. II [4]**	**Kurz et al. [5]**
**Analyte scope**	Glyphosate, AMPA, NAGly, glufosinate, MPPA, NAGlu, fosetyl, ethephon, HEPA, cyanuric acid, chlorate, perchlorate, phosphonic acid, bromide, trifluoroacetic acid	Glyphosate, AMPA, *N*-acetyl-AMPA, glufosinate, MPPA, NAGlu, fosetyl, ethephon, cyanuric acid, chlorate, perchlorate, phosphonic acid, clopyralid, bialaphos	Glyphosate, AMPA, *N*-acetyl-AMPA, NAGly, fosetyl, chlorate, perchlorate, phosphonic acid	Ethephon, HEPA, chlorate, perchlorate	Fosetyl, phosphonic acid	Glyphosate, AMPA, glufosinate, fosetyl, clopyralid
**Use of IL-IS**	all, except bromide	Glyphosate, glufosinate, MPPA, NAGlu, cyanuric acid, chlorate, perchlorate	NAGly	Ethephon, chlorate, perchlorate	Phosphonic acid	-
**Matrix scope**	Plant and animal origin	Plant origin	Plant origin	Plant origin	Plant origin	Water
**IC column and elution**	AS19 and AS24, hydroxide elution	AS19, hydroxide elution	AS19 and AS11, hydroxide elution	Metrosep A, bicarbonate in water/ACN elution	Metrosep A, bicarbonate in water/ACN elution	AS24, hydroxide elution
**Chromato-graphy system**	IC with suppressor	IC with suppressor	IC with suppressor	LC without suppressor	LC without suppressor	IC with suppressor
**MS detection**	Triplequad	Triplequad	Orbitrap	Triplequad	Triplequad	Triplequad
**Make-up solvent**	ACN at 1:2 of IC-flow	ACN at 1:2.75 of IC-flow	ACN at 1:1 of IC-flow	-	-	2-propanol at 1:3 of IC-flow

Table S2: *IL-IS used, concentration in spiking solutions, of which 100 µL were added prior to extraction in validation experiments, concentration in 10 g and 5 g sample portion and measured mass transitions in MS/MS, see also [1].*

Compound	Concentration in spiking solutions in µg/mL	Concentration in 10 g sample portion in mg/kg	Concentration in 5 g sample portion in mg/kg	Mass transition
**Glyphosate $^{13}C_2$ ^{15}N**	20	0.2	0.4	*m/z* 171 → 63
**AMPA ^{13}C ^{15}N**	40	0.4	0.8	*m/z* 112 → 63
***N*-Acetyl-Glyphosate (NAGly) $^{13}C_2$ ^{15}N**	20	0.2	0.4	*m/z* 213 → 63
**Fosetyl D_5**	20	0.2	0.4	*m/z* 114 → 82
**Ethephon D_4**	20	0.2	0.4	*m/z* 147 → 111
**HEPA D_4**	20	0.2	0.4	*m/z* 129 → 79
**Glufosinate D_3**	20	0.2	0.4	*m/z* 183 → 63
**MPPA D_3**	20	0.2	0.4	*m/z* 154 → 63
***N*-Acetyl-Glufosinate (NAGlu) D_3**	20	0.2	0.4	*m/z* 225 → 63
**Cyanuric acid $^{13}C_3$**	20	0.2	0.4	*m/z* 131 → 43
**Chlorate $^{18}O_3$**	20	0.2	0.4	*m/z* 89 → 71
**Perchlorate $^{18}O_4$**	20	0.2	0.4	*m/z* 107 → 89
**Phosphonic acid $^{18}O_3$**	20	0.2	0.4	*m/z* 87 → 85
**Trifluoroacetic acid $^{13}C_2$**	10	0.1	0.2	*m/z* 115 → 70

Table S3: *IC-MS/MS and LC-MS/MS conditions employed in this study.*

**IC-MS/MS conditions [1,2]**		
**Column/Pre-column**	Thermo Scientific Dionex IonPac AS19 2x 250 mm with AG19 2x 50 mm and AS24 2x 250 mm with AG24 2x 50mm	
**Potassium hydroxide (KOH) gradient for separation**	**Time**	**Molarity of KOH in mmol/L**
	0	15
	8	15
	13	36
	21	36
	21.5	70
	25	70
	25.5	15
	30	15
**Flow rate (IC)**	0.3 mL/min	
**Injection volume**	5 µL of 5-fold diluted extracts in ultrapure water	
**Eluent(source)**	Thermo Scientific Dionex EGC 500 KOH eluent generator cartridge	
**Suppressor**	Dionex ASRS 300; 2mm	
**Temperature**	Column oven: 32 °C, Suppressor: 15 °C	
**Flow rate (of make-up solvent)**	0.15 mL/min ACN (MS-grade)	
**Ion source**	ESI Turbo V ion source, negative mode	
**Curtain gas (nitrogen) pressure**	30 psi	
**Ion Spray Voltage**	-4500 V	
**Gas supply**	Gas 1 (nebulizer): 60 psi; Gas 2 (heater): 60 psi	
**Temperature of Gas 2**	600 °C	
**LC-MS/MS Conditions HILIC/Torus: Method M1.6 of QuPPe-PO-Method [1]**		
**LC-MS/MS Conditions PGC/Hypercarb: Method M1.3 of QuPPe-PO-Method [1]**		

Table S4: *Mass transitions of the analytes recorded with MS/MS, see also [1].*

**Compound**	**Mass Transition 1 (target)**	**Mass Transition 2**	**Mass Transition 3**
**Glyphosate**	*m/z* 168 → 63	*m/z* 168 → 150	*m/z* 168 → 124
**AMPA**	*m/z* 110 → 63	*m/z* 110 → 79	*m/z* 110 → 81
**NAGly**	*m/z* 210 → 63	*m/z* 210 → 150	*m/z* 210 → 124
**Fosetyl**	*m/z* 109 → 81	*m/z* 109 → 63	*m/z* 109 → 79
**Ethephon**	*m/z* 143 → 107	*m/z* 145 → 107	*m/z* 143 → 79
**HEPA**	*m/z* 125 → 63	*m/z* 125 → 95	*m/z* 125 → 107
**Glufosinate**	*m/z* 180 → 63	*m/z* 180 → 95	*m/z* 180 → 85
**MPPA**	*m/z* 151 → 63	*m/z* 151 → 133	*m/z* 151 → 107
**NAGlu**	*m/z* 222 → 63	*m/z* 222 → 136	*m/z* 222 → 59
**Cyanuric acid**	*m/z* 128 → 42	*m/z* 128 → 85	
**Chlorate**	*m/z* 83 → 67	*m/z* 85 → 69	*m/z* 85 → 67
**Perchlorate**	*m/z* 99 → 83	*m/z* 101 → 85	*m/z* 99 → 67
**Bromide****	*m/z* 81 → 81	*m/z* 79 → 79	
**Phosphonic acid**	*m/z* 81 → 79	*m/z* 81 → 63	
**Trifluoroacetic acid**	*m/z* 113 → 69	*m/z* 113 → 113*	

*no other transition available

** high collision energy was used [1]

Table S5: *Overview of retention times and contents of exemplary anionic or potentially anionic matrix components in lemon, soybean, rhubarb, Swiss chard and cucumber according to literature [3] in mg/100g and concentrations in undiluted QuPPe extracts in mg/mL (assuming quantitative extraction). Values were calculated for 10 g sample, except for soybean, where 5 g were used.*

Matrix component	Retention time*	Lemon		Soybean		Rhubarb	
		Literature [mg/100g]	QuPPe extract [mg/mL]	Literature [mg/100g]	QuPPe extract** [mg/mL]	Literature [mg/100g]	QuPPe extract [mg/mL]
**Citric acid**	25.3	4683	23.4			130	0.7
**Malic acid**	15.5	200	1.0			1250	6.3
**Phosphate**	19.8			550	1.4	22	0.1
**Nitrate**	10.9					215	1.1
**Chloride**	6.1					60	0.3
**Sulfate**	14.5						
**Oxalic acid**	16.1					270	1.4

Matrix component	Retention time*	Swiss chard		Cucumber	
		Literature [mg/100g]	QuPPe extract [mg/mL]	Literature [mg/100g]	QuPPe extract [mg/mL]
**Citric acid**	25.3			20	0.1
**Malic acid**	15.5			240	1.2
**Phosphate**	19.8	39	0.2	15	0.1
**Nitrate**	10.9	487	2.4	19	0.1
**Chloride**	6.1			37	0.2
**Sulfate**	14.5				
**Oxalic acid**	16.1				

*Using ion chromatography on a AS19 column, and detection via conductivity

** using 5 g analytical portion

Table S6: *Full table of effect of the make-up solvents ACN, methanol, 2-propanol at ratios of ~1:4 to ~5:4 compared to a constant IC flow rate of 0.3 mL/min. The % value of the peak areas were compared with injections in pure water (no make-up solvent), which was set at 100%. Each measurement was performed in triplicate and mean values (and relative standard deviation (RSD) in brackets) were given for the most prominent MS/MS transitions. The best values are highlighted in bold letters.*

Compound	Solvent	Flow-rate of make-up solvent (external pump)*				
		**0.08 mL**	**0.15 mL**	**0.23 mL**	**0.3 mL**	**0.38 mL**
		Make-up solvent flow-rate compared to IC effluent flow-rate (1:1 = equal)				
		**~1:4**	**2:4 (1:2)**	**~3:4**	**4:4 (1:1)**	**~5:4**
		Share of make-up solvent on total flow after admixture				
		**20%**	**33%**	**43%**	**50%**	**56%**
		Normalized peak areas (no make-up solvent set at 100%); RSD in % in brackets				
**Glyphosate**	Acetonitrile	**189%** (7%)	**180%** (6%)	**197%** (5%)	175% (1%)	162% (3%)
	Methanol	**153%** (5%)	123% (5%)	103% (2%)	86% (7%)	71% (7%)
	2-Propanol	100% (19%)	**150%** (10%)	86% (1%)	87% (3%)	79% (4%)
**AMPA**	Acetonitrile	**196%** (4%)	168% (3%)	**201%** (4%)	**186%** (5%)	175% (6%)
	Methanol	**144%** (4%)	117% (4%)	107% (5%)	86% (1%)	65% (15%)
	2-Propanol	105% (13%)	**131%** (5%)	98% (14%)	88% (9%)	100% (7%)
**NAGly**	Acetonitrile	**223%** (5%)	169% (3%)	181% (12%)	138% (2%)	146% (7%)
	Methanol	**236%** (7%)	**232%** (3%)	**248%** (9%)	201% (12%)	152% (11%)
	2-Propanol	112% (20%)	**182%** (11%)	124% (10%)	136% (6%)	125% (8%)
**Fosetyl**	Acetonitrile	206% (5%)	**224%** (3%)	**236%** (3%)	**225%** (8%)	**216%** (5%)
	Methanol	**150%** (7%)	**156%** (1%)	**163%** (1%)	**152%** (1%)	132% (4%)
	2-Propanol	55% (29%)	76% (21%)	95% (19%)	**114%** (12%)	**109%** (18%)
**Ethephon**	Acetonitrile	**181%** (3%)	**179%** (2%)	**177%** (1%)	**167%** (3%)	146% (0.1%)
	Methanol	136% (17%)	**163%** (2%)	**161%** (2%)	**153%** (2%)	114% (8%)
	2-Propanol	97% (20%)	**189%** (8%)	118% (7%)	125% (14%)	101% (11%)
**HEPA**	Acetonitrile	**170%** (2%)	**169%** (4%)	**180%** (4%)	**175%** (2%)	160% (3%)
	Methanol	139% (5%)	**151%** (4%)	133% (4%)	117% (4%)	99% (6%)
	2-Propanol	105% (17%)	**182%** (10%)	136% (4%)	144% (9%)	132% (10%)
**Glufosinate**	Acetonitrile	**146%** (4%)	113% (2%)	116% (6%)	96% (4%)	84% (12%)
	Methanol	**126%** (5%)	105% (4%)	103% (7%)	87% (7%)	68% (13%)
	2-Propanol	101% (19%)	**158%** (10%)	127% (8%)	133% (2%)	134% (6%)
**MPPA**	Acetonitrile	**164%** (6%)	**153%** (3%)	**161%** (4%)	**147%** (2%)	141% (7%)
	Methanol	121% (9%)	**134%** (4%)	**136%** (3%)	123% (2%)	103% (7%)
	2-Propanol	93% (18%)	**155%** (9%)	91% (3%)	94% (11%)	82% (9%)

**NAGlu**	Acetonitrile	**216%** (5%)	141% (4%)	136% (9%)	112% (10%)	99% (16%)
	Methanol	208% (6%)	267% 0.2%)	**380%** (6%)	**346%** (9%)	263% (8%)
	2-Propanol	118% (13%)	**183%** (11%)	147% (2%)	154% (5%)	147% (7%)
**Chlorate**	Acetonitrile	**200%** (8%)	**194%** (4%)	**209%** (2%)	**209%** (4%)	**208%** (5%)
	Methanol	179% (7%)	216% (3%)	**276%** (3%)	**262%** (2%)	**249%** (3%)
	2-Propanol	124% (22%)	**239%** (5%)	197% (2%)	205% (8%)	184% (1%)
**Perchlorate**	Acetonitrile	**434%** (6%)	299% (14%)	251% (8%)	217% (5%)	219% (2%)
	Methanol	**339%** (6%)	**329%** (13%)	**326%** (13%)	218% (14%)	158% (9%)
	2-Propanol	169% (11%)	**241%** (2%)	178% (3%)	188% (6%)	179% (5%)
**Phosphonic**	Acetonitrile	**188%** 0.3%)	**186%** (1%)	**190%** (3%)	170% (5%)	158% (3%)
**Acid**	Methanol	**182%** (9%)	**190%** (4%)	169% (1%)	141% (3%)	114% (9%)
	2-Propanol	68% (18%)	89% (22%)	105% (16%)	**122%** (6%)	**114%** (15%)

* the flow-rate of AXP-MS pump was only adjustable to two decimals. Therefore, increments derived from the standard value of 0.3 mL (i.e. identical with the flow rate of the mobile phase) were rounded to two decimals)

Table S7: *Validation data of target transitions in strawberry and milk using IC-MS/MS in comparison to LC-MS/MS (PGC and HILIC) (validated levels do not represent lowest successfully validated levels). Recoveries were determined using external matrix-matched calibrations using matching isotopically labelled standards for each analyte.*

					**IC (AS19)**		**LC HILIC (Torus DEA)**		**LC PGC (Hypercarb)**	
Analyte	Mass transition	Matrix	Validated Level in mg/kg	No. of repli-cates	Average Recovery (%)	RSD (%)	Average Recovery (%)	RSD (%)	Average Recovery (%)	RSD (%)
**AMPA**	*m/z* 110 → 63	**Strawberry**	0.05	5	107	2.5	109	4.3	107	5.4
			0.1	5	102	2.1	101	1.8	98	5.1
**Cyanuric acid**	*m/z* 128 → 42		0.05	5	97	6.5	*	*	104	3.7
			0.1	5	101	8.3	*	*	98	4.0
**Ethephon**	*m/z* 143 → 107		0.01	5	95	12	99	9.5	99	7.6
			0.02	5	95	5.4	94	6.3	100	5.9
**Fosetyl**	*m/z* 109 → 81		0.01	5	98	3.4	107	1.8	102	4.2
			0.02	5	99	2.1	101	1.9	100	1.5
**Glufosinate**	*m/z* 180 → 63		0.03	5	101	3.7	99	9.7	102	0.9
			0.06	5	100	1.5	97	5.0	98	3.0
**Glyphosate**	*m/z* 168 → 63		0.05	5	98	8.8	99	3.7	103	2.1
			0.1	5	97	4.7	93	7.0	99	1.4
**HEPA**	*m/z* 125 → 63		0.02	5	104	15	110	5.0	67	20
			0.04	5	102	3.9	97	4.2	92	5.4
**MPPA**	*m/z* 151 → 63		0.02	5	97	5.6	102	8.6	102	3.1
			0.04	5	103	3.8	94	4.2	98	2.6
**NAGlu**	*m/z* 222 → 63		0.02	5	108	4.3	122	2.9	105	1.4
			0.04	5	100	1.5	99	6.7	100	1.3
**NAGly**	*m/z* 210 → 63		0.05	5	99	4.2	108	3.2	104	1.7
			0.1	5	103	5.1	100	2.0	101	0.9
**AMPA**	*m/z* 110 → 63	**Milk**	0.05	5	97	3.8	99	6.6	102	4.2
**Cyanuric acid**	*m/z* 128 → 42		0.05	5	98	12	*	*	111	8.5
**Ethephon**	*m/z* 143 → 107		0.01	5	94	5.9	107	7.3	***	***
**Fosetyl**	*m/z* 109 → 81		0.01	5	97	1.4	108	3.3	96	2.6
**Glufosinate**	*m/z* 180 → 63		0.03	5	96	2.8	92	5.9	100	5.7
**Glyphosate**	*m/z* 168 → 63		0.05	5	96	3.8	100	1.0	102	3.9
**HEPA**	*m/z* 125 → 63		0.02	5	100	7.1	97	7.4	106	8.7
**MPPA**	*m/z* 151 → 63		0.02	5	97	1.5	102	5.4	97	2.7
**NAGlu**	*m/z* 222 → 63		0.02	5	95	4.1	99	7.5	97	4.9
**NAGly**	*m/z* 210 → 63		0.05	5	97	3.6	101	6.9	95	5.9
**Phosphonic acid**	*m/z* 81 → 79		0.05	5	94	2.3	90	10	**	**
			0.1	5	101	1.8	93	11	**	**
**Chlorate**	*m/z* 85 → 51		0.03	5	86	6.0	72	13	**	**
			0.06	5	100	1.3	94	4.2	**	**
**Perchlorate**	*m/z* 101 → 85		0.01	5	102	5.2	**	**	**	**
			0.02	5	104	1.7	**	**	**	**
**Bromide**	*m/z* 79 → 79		5	5	98	3.8	85	3.7	**	**
			10	5	101	6.4	92	4.9	**	**
**Trifluoracetic acid**	*m/z* 113 → 69		0.025	5	103	21.8	***	***	*	*
			0.05	5	102	1.3	***	***	*	*

* not included in this method
** not measured
*** validation at that level not successful

Table S8: *Number of positive findings in routine analysis of incurred residues in market samples, using IC-MS/MS (AS19) and LC-MS/MS (Hypercarb) by analyte.*

**Analyte**	**IC-MS/MS**			**LC-MS/MS PGC (Hypercarb)**		
	LOQ [mg/kg]*	>LOQ	<LOQ***	LOQ [mg/kg]*	>LOQ	<LOQ***
**AMPA**	0.01	0	0	0.01	0	2
**Cyanuric acid**	0.05	8	0	0.005	31	11
**Ethephon**	0.01	2	0	0.01	1	3
**Fosetyl**	0.01	1	2	0.01	1	4
**Glufosinate**	0.01	0	3	0.01	0	0
**Glyphosate**	0.01	0	5	0.02	0	1
**HEPA**	0.01	0	6	0.01	2	0
**MPPA**	0.01	1	3	0.01	2	2
**NAGlu**	0.02	0	2	0.02	1	0
**NAGly**	0.02	0	0	0.02	0	0
**Bromide****	5	1	21	5	0	19
**Chlorate**	0.005	27	1	0.01	15	20
**Perchlorate**	0.005	36	8	0.01	17	19
**Phosphonic acid**	0.01	76	13	0.05	32	17
**Trifluoracetic acid**	0.02	31	30	-	-	-

*the LOQ indicated here refers to fruits and vegetables and refers to the lowest successfully validated level

**the LOQ was arbitrarily set at 5 mg/kg due to the natural background levels of bromide; values "<LOQ" were in the range between >0.5 and <5 mg/kg

*** Identified analytes at semiquantitative levels (<LOQ, but typically not lower than 10%LOQ)

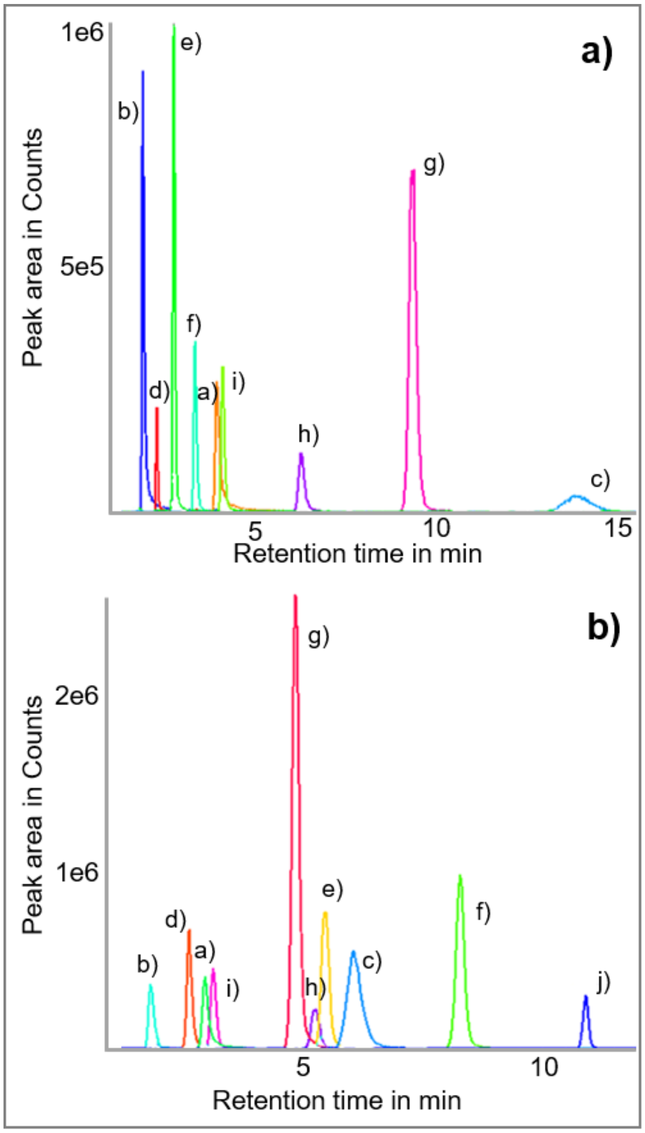

Fig. S1: *LC/MSMS chromatograms of ten pesticide standards* ***(a)*** *on a Torus DEA (HILIC) column and (**b**) a Hypercarb (PGC) column. Compounds: a) glyphosate, b) AMPA, c) NAGly, d) glufosinate, e) MPPA, f) NAGlu, g) fosetyl, h) ethephon, i) HEPA, j) cyanuric acid, each at 0.1 µg/mL in solvent*

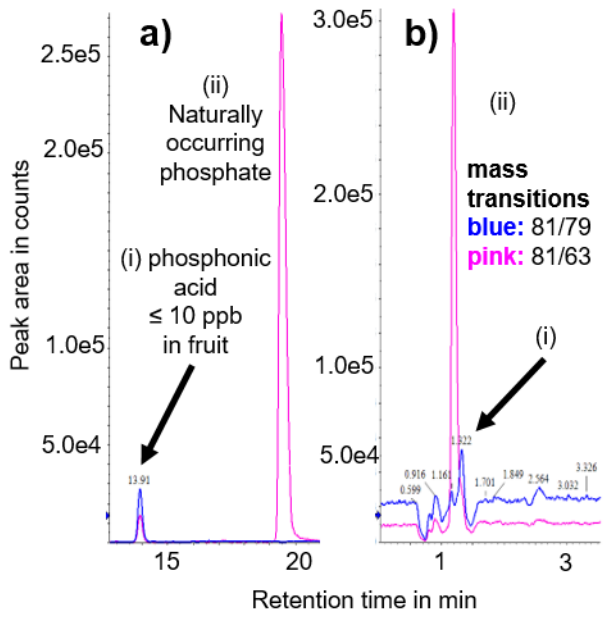

Fig. S2: ***(a)*** *IC-MS/MS (AS19; 5-fold diluted QuPPe extract) and* ***(b****) LC-MS/MS (PGC; 10-fold diluted QuPPe extract) chromatograms of phosphonic acid and phosphate in an extract of a fruit preparation for infants with phosphonic acid being contained at a level ~ 0.01 mg/kg and natural contents of phosphate. While the target transition of phosphonate (m/z 81 → 79) is largely unaffected by phosphate, the qualifier transition (m/z 81 → 63) is strongly interfered by phosphate and therefore useless in case of a partial co-elution of phosphate and phosphonate. The resolution on the PGC column can vary depending on the column condition. The particular example represents an extreme case, combining not only a very poor resolution, but also a very low phosphonate level and the presence of phosphate at very high levels*

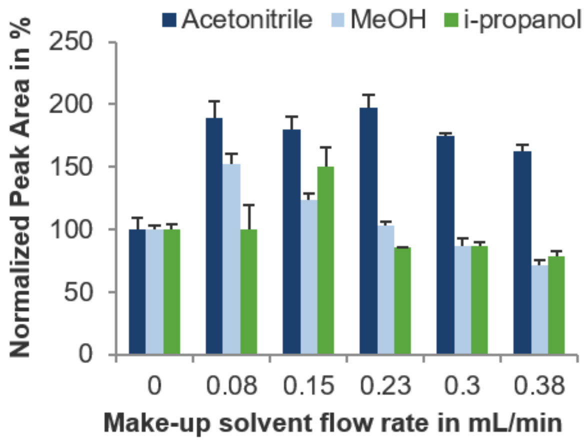

Fig. S3*: Average normalized peak areas (n=3) of glyphosate according to the applied flow rate of the make-up pump. Values measured without the make-up pump (flow rate 0 mL/min) function as reference and are set at 100%*

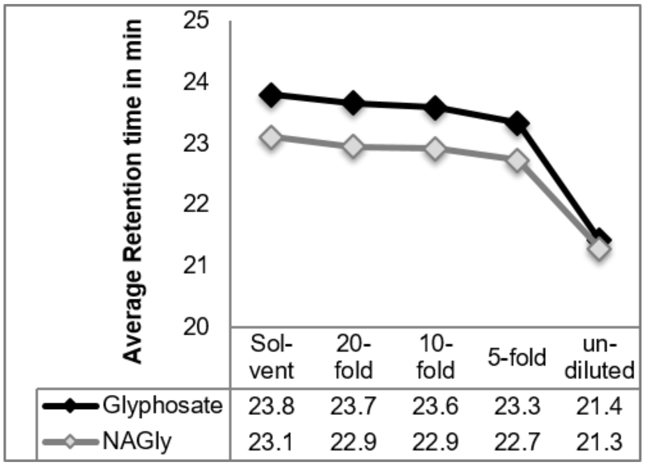

Fig. S4: *Average retention times (t_R in min) of glyphosate and NAGly in undiluted and differently diluted lemon extracts (n=3)*

References

[1] Adams S, Guest J, Dickinson M, Fussell RJ, Beck J, Schousten F. Development and validation of ion chromatography-tandem mass spectrometry-based method for the multiresidue determination of polar ionic pesticides in food. J Agr Food Chem. 2017;65(34):7294 – 7304.

[2] Rajski L, Galiano FJD, Cutillas V, Fernández-Alba AR. Coupling ion chromatography to Q-orbitrap for the fast and robust analysis of anionic pesticides in fruits and vegetables. J AOAC Int. 2018;101(2):352-359.

[3] Bauer A, Luetjohann J, Rohn S, Kuballa J, Jantzen E. Ion chromatography tandem mass spectrometry (IC-MS/MS) multimethod for the determination of highly polar pesticides in plant-derived commodities. Food Control. 2018;86:71-76.

[4] Bauer A, Luetjohann J, Rohn S, Kuballa J, Jantzen E. Determination of fosetyl and phosphonic acid at 0.010 mg/kg level by ion chromatography tandem mass spectrometry. J Agr Food Chem. 2018;66(1):346-350.

[5] Kurz A, Bousova K, Beck J, Schoutsen, Bruggink C, Kozeluh, Kule L, Godula M. Routine analysis of polar pesticides in water at low ng/L levels by ion chromatography coupled to triple quadrupole mass spectrometer. Application note 666. 2017. https://lcms.cz/labrulez-bucket-strapi-h3hsga3/AN_666_IC_MS_Polar_Pesticides_Water_AN_64945_EN_e84581b9a7/AN-666-IC-MS-Polar-Pesticides-Water-AN64945-EN.pdf. Accessed 10 Apr 2024.

[6] Anastassiades M, Wachtler A-K, Kolberg DI, Eichhorn E, Marks H, Benkenstein A, Zechmann S, Mack D, Wildgrube C, Barth A, Sigalov I, Goerlich S, Dörk D, Cerchia G. Quick Method for the Analysis of Highly Polar Pesticides in Food Involving Extraction with Acidified Methanol and LC- or IC-MS/MS Measurement I. Food of Plant Origin (QuPPe-PO-Method), Version 12.2, 21.12.2023. https://www.eurl-pesticides.eu/userfiles/file/EurlSRM/ EurlSrm_meth_QuPPe_PO_V12_2.pdf. Accessed 10 Apr 2024.

[7] Wachtler A-K, Wildgrube C, Mack D, Barth, Anastassiades M, Scherbaum E, Vetter W. Analysis of Highly Polar Pesticides in Food of Plant and Animal Origin by IC-MS/MS: 13th EPRW 2020, PD-87: https://www.eurl-

pesticides.eu/userfiles/file/EurlSRM/EPRW%202020%20-%20PD87.pdf. Accessed 10 Apr 2024.

[8] Souci, Fachmann, Kraut: Food composition and nutrition table. Online Database. Accessed 10 Apr 2024.
